# A Protein Prioritization Approach Tailored for the FA/BRCA Pathway

**DOI:** 10.1371/journal.pone.0062017

**Published:** 2013-04-19

**Authors:** Anneke Haitjema, Bernd W. Brandt, Najim Ameziane, Patrick May, Jaap Heringa, Johan P. de Winter, Hans Joenje, Josephine C. Dorsman

**Affiliations:** 1 Department of Clinical Genetics, VU University Medical Center, Amsterdam, The Netherlands; 2 Centre for Integrative Bioinformatics VU (IBIVU), VU University Amsterdam, Amsterdam, The Netherlands; 3 Department of Preventive Dentistry, Academic Centre for Dentistry Amsterdam (ACTA), University of Amsterdam and VU University Amsterdam, Amsterdam, The Netherlands; 4 Luxembourg Centre for Systems Biomedicine, University of Luxembourg, Esch-sur-Alzette, Luxembourg; 5 Institute for Systems Biology, Seattle, Washington, United States of America; CHA University, Republic of Korea

## Abstract

Fanconi anemia (FA) is a heterogeneous recessive disorder associated with a markedly elevated risk to develop cancer. To date sixteen FA genes have been identified, three of which predispose heterozygous mutation carriers to breast cancer. The FA proteins work together in a genome maintenance pathway, the so-called FA/BRCA pathway which is important during the *S* phase of the cell cycle. Since not all FA patients can be linked to (one of) the sixteen known complementation groups, new FA genes remain to be identified. In addition the complex FA network remains to be further unravelled. One of the FA genes, *FANCI*, has been identified via a combination of bioinformatic techniques exploiting FA protein properties and genetic linkage. The aim of this study was to develop a prioritization approach for proteins of the entire human proteome that potentially interact with the FA/BRCA pathway or are novel candidate FA genes. To this end, we combined the original bioinformatics approach based on the properties of the first thirteen FA proteins identified with publicly available tools for protein-protein interactions, literature mining (Nermal) and a protein function prediction tool (FuncNet). Importantly, the three newest FA proteins FANCO/RAD51C, FANCP/SLX4, and XRCC2 displayed scores in the range of the already known FA proteins. Likewise, a prime candidate FA gene based on next generation sequencing and having a very low score was subsequently disproven by functional studies for the FA phenotype. Furthermore, the approach strongly enriches for GO terms such as DNA repair, response to DNA damage stimulus, and cell cycle-regulated genes. Additionally, overlaying the top 150 with a haploinsufficiency probability score, renders the approach more tailored for identifying breast cancer related genes. This approach may be useful for prioritization of putative novel FA or breast cancer genes from next generation sequencing efforts.

## Introduction

Fanconi anemia (FA) is a rare recessive genetically heterogeneous chromosomal instability disorder with both autosomal and X-linked inheritance. FA is associated with haematological defects, including bone marrow failure, aplastic anemia, myelodysplastic syndrome (MDS) and childhood acute myeloid leukemia (AML). Besides these problems, patients have a high risk for solid tumours, such as head and neck squamous cell carcinoma, gynaecological squamous cell carcinoma and esophageal carcinoma. In addition, the patients can present with liver tumours, skin tumours, brain tumours, and renal tumours [1]–[4]. The high cancer risk is generally attributed to impaired repair of DNA damage. Cells from FA patients display chromosomal structural abnormalities. Accordingly, patient-derived cells have turned out to be extremely sensitive to bifunctional alkylating or DNA interstrand cross-linking agents, such as mitomycin C or cisplatin [5], [6]. The latter feature is currently used in standard FA diagnostics.

**Figure 1:**
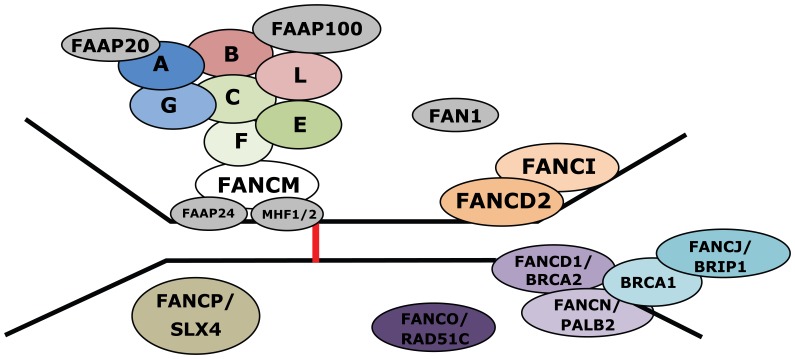
The Fanconi anemia pathway. For further explanation see main text (newest FA member XRCC2 not shown).

Until now, fifteen complementation groups, FA-A, -B, -C, -D1, -D2, -E, -F, -G, -I, -J, -L, -M, -N, -O, and -P have been described, each corresponding to one distinct gene causing FA [7]–[28]. Recently, the sixteenth novel FA group was found to have mutations in XRCC2 [29]. The FA proteins function together in the FA/BRCA pathway, in which the monoubiquitination of FANCD2 and FANCI is the central event. This ubiquitination reaction is catalyzed by the so-called FA core complex, which consists of eight FA proteins (FANC-A, -B, -C, -E, -F, -G, -L, and -M). The activation of FANCD2 and FANCI recruits the other factors of the pathway, FANCD1/BRCA2, FANCJ/BRIP1, FANCN/PALB2, FANCO/RAD51C, FANCP/SLX4, and XRCC2 to repair DNA damage (Figure 1). Beside these sixteen FA proteins, there are several other proteins associating with the FA core complex, but no mutations in the corresponding genes have thus far been found. These proteins are the Fanconi Anemia Associated Proteins (FAAPs): FAAP100, FAAP24, FAAP20, FAAP16/MHF1, and FAAP10/MHF2 [30]–[33]. A FANCD2/FANCI associated nuclease, FAN1, was also recently identified [34]–[37], as well as a deubiquitinating enzyme complex consisting of USP1 and UAF1 [38], [39]. Intriguingly, most FA proteins were orphan proteins at the time of their discovery, showing no homology to other proteins and harbouring few known protein domains, while displaying a moderate evolutionary conservation. Since not all FA patients could be linked to (one of) the sixteen known complementation groups, new FA genes remain to be identified. In addition, the complex FA network remains to be further unravelled. In a previous study, bioinformatics, based on the properties of known FA proteins, was successfully combined with genetic linkage in identifying a novel FA gene, *FANCI* from a candidate list [18].

The aim of this study is to develop an approach for the identification of proteins of the entire human proteome that potentially interact with the FA/BRCA pathway or are candidate novel FA genes. To this end, we combined a bioinformatic approach based on properties of the first thirteen identified FA proteins with publicly available tools allowing protein ranking.

## Materials and Methods

The FA proteins play a pivotal role in genome maintenance, especially during the *S* phase of the cell cycle (Figure 1). The proteins of the so-called FA core complex monoubiquitinate FANCD2/FANCI resulting in activation of the downstream operating FA proteins. To generate a prioritization of candidate FA/FA-interacting proteins, an integrated approach was developed that involved screening of the entire human proteome (EnsEMBL) using multiple publicly available bioinformatic tools and databases. One of the sixteen known FA genes, *FANCI*, was discovered via selection of genes based on (general) FA protein properties from a limited number of ∼300 candidate genes [18]. This bioinformatics strategy was incorporated in the selection scheme, using the information of the first thirteen identified FA proteins available at the time. This information was combined with data from publicly available bioinformatic tools and databases.

The entire proteome was scanned for peptides with intrinsic properties that are shared by the first thirteen identified FA proteins (FANC-A, -B, -C, -D1, -D2, -E, -F, -G, -I, -J, -L, -M, and -N). The pipeline analysis started with the calculation of nuclear localization scores (WoLF PSORT; [40]) and iso-electric points (EMBOSS IEP; [41]). Orthology/paralogy relationships to mouse were obtained via EnsemblCompara [42], and Gene Ontology (GO [43]; from EnsEMBL), protein interaction data (EnVision 2), biosemantics (Nermal; [44]) and protein function analysis (FuncNet; including the literature mining tool iHop; [45]) were used. The combined information was used for ranking.

### Data sources and Tools

All 77454 peptides of the human protein-coding genes were downloaded from EnsEMBL (version 56) using Biomart, together with their corresponding EnsEMBL protein, transcript, and gene IDs (ENSP, ENST, and ENSG). The peptides were used to score the 22413 associated genes.

For all protein-coding genes, the associated gene name and database, gene description, Online Mendelian Inheritance in Man (OMIM) morbid and gene accession, and GO cellular component terms were downloaded. The human data were linked to data on mouse. Human to mouse and mouse to human amino acid percentage identity, orthology type and representative (human) protein IDs were retrieved from ComparaGene. The corresponding FASTA sequences of the human proteins were used to calculate the peptide length, iso-electric point (pI; [41]), and Nuclear Localization Signal (NLS; [40]). The GO cellular component annotations for the proteins were analyzed for hierarchic relationships to nucleus (GO:0005634) with the SOAP interface to the Ontology Lookup Service of the European Bioinformatics Institute [46], [47].

Thirteen FA proteins have been used to find interacting proteins using the Molecular Interactions workflow of EnVision 2, Nermal (biosemantics), and FuncNet. The UniProt IDs of the FA proteins have been used as input to the EnVision 2 Molecular Interaction workflow to retrieve interaction data. All thirteen FA proteins were queried against: ChEMBL, DIP, IntAct, iRefIndex, MatrixDB, MINT, MPIDB, Reactome, and Reactome-Functional-Interactions (STRING and BioGrid were not available at the time). Databases that returned output were: DIP, IntAct, iRefIndex, MINT, and Reactome. Next, all UniProt/RefSeq IDs were taken and mapped to EnsEMBL peptide IDs with the Protein Identifier Cross-Reference service (PICR; [48]).

Nermal is a text-mining tool that predicts protein-protein interaction based on the similarity of the context in which proteins appear in the literature [44]. The entire matrix with the full set of all human protein-pair match scores was downloaded from the Nermal website. Since we used thirteen FA proteins, each query protein can have interactions with more than one FA protein. In case multiple interactions are present with the FA cluster, the maximum score of the query protein with any of these thirteen FA proteins was taken. Proteins interacting with one of the thirteen FA proteins according to Nermal were listed. Further, FuncNet, a web-based protein function prediction tool [45] was used to give prediction scores to the whole proteome. The thirteen FA proteins were used as reference set while the entire human proteome was divided over several query sets. The FuncNet and Nermal scores were integrated and a new Fisher score was calculated that includes all FuncNet P-values as well as a P-value for Nermal.

## Results and Discussion

The FA pathway consists of sixteen FA proteins and many associating proteins. However, still unclassified FA patients exist, which have no gene defect in any of the described complementation groups or in the associating genes/proteins. This suggests that there are more genes/proteins involved in this pathway. Since the unclassified patient groups are very small or even consist of a single person, discovery of the gene by linkage analysis is hard. Modern techniques, such as whole genome sequencing of DNA from unclassified FA families, result in many candidate genes. Therefore, to further elaborate which proteins participate in the FA pathway, we used the pathway itself to screen the whole proteome for similar proteins.

### Properties of FA Proteins

During the identification of *FANCI* the protein properties of existing FA proteins were used to narrow down the candidate list generated by genetic linkage [18]. Since we could use protein properties to distinguish more likely candidates we decided for a genome wide approach. We first compared the intrinsic protein properties of thirteen established FA proteins in order to determine several ‘common’ FA ranges.

The following protein properties have been compared between the first thirteen identified FA proteins and the latest identified FA proteins, FANCO/RAD51C, FANCP/SLX4, and XRCC2: i) human-mouse conservation (%ID aa); ii) Nuclear Localization Signal (NLS) score; iii) iso-electric points (pI); iv) subcellular localization, and v) mRNA expression patterns in normal tissues (Table 1; first 4 criteria, all sixteen described FA proteins).

**Table 1:** Overview of the main intrinsic protein properties of sixteen FA proteins.

	Percentage identity (aa)					
Gene	Human-Mouse	Mouse-Human	NLS score	pI	Length (aa)	Cellular localization
FANCA	65	66	0.86	6.57	1455	N/C
FANCG	72	72	−0.47	5.13	622	N/C
FANCB	49	49	0.95	7.65	859	N
FANCL	78	79	−0.47	6.42	375	N/C
FANCC	67	63	−0.47	6.09	558	N/C
FANCE	65^*^EnsEMBL:41	66^*^EnsEMBL:53	0.70	4.89	536	N
FANCF	47^*^EnsEMBL:N/A	51^*^EnsEMBL:N/A	−0.47	9.20	374	N
FANCM	63	64	0.76	6.00	2048	N
FANCD2	73	74	−0.03	6.19	1471	N
FANCI	80	80	1.70	6.72	1328	N
FANCD1/BRCA2	56	58	1.02	6.70	3418	N
FANCN/PALB2	58	63	0.76	6.41	1186	N
FANCJ/BRIP1	69	74	1.13	6.91	1249	N
FANCO/RAD51C	76	75	−0.47	6.73	376	N
FANCP/SLX4	51	60	4.91	5.95	1834	N
XRCC2	77	78	−0.47	5.95	280	N

NLS score: Nuclear Localization Signal score. pI: iso-electric point. Cellular localization: N = Nucleus; N/C = Nucleus and Cytoplasm. ^*^These percentages have been calculated using RefSeq sequences as the orthologs assignment in EnsEMBL were incorrect. FANCE: EnsEMBL’s ortholog mouse protein incorrect; FANCE: NP_068741 (human: 100% identical with ENSP00000229769) and NP_001157291 (mouse). FANCF: no mouse ortholog available in EnsEMBL. FANCF: NP_073562.1 (human: 100% identical with ENSP00000330875) and NP_001108559.1 (mouse).

As described previously [18], a significant subset of FA genes encoded orphan proteins at the time of their discovery. The mouse orthologs of the FA proteins display between ∼50 to ∼80% amino acid identity with the corresponding human protein. FANCF has the lowest conservation with an amino acid identity of 47% with its mouse ortholog, FANCI the highest with 80%, while the average for 16 FA proteins is 65% (Table 1). With WoLF PSORT, a subcellular localization prediction has been generated based on the amino acid sequences. FA proteins with a positive NLS score are: FANC-A, -B, -E, -M, -I, -D1, -N, -J, and -P, with an average score of 1.42 (a score of NLS >1 means a predicted specificity of 71% of being nuclear). The FA core complex is assembled from subcomplexes [49], and intriguingly always one member of the subcomplex has a positive NLS score. The score of the most recently identified member of the FA protein family, FANCP/SLX4, turned out to be high (4.91) in comparison to other FA proteins. This may suggest that the prime function of FANCP/SLX4 is in the nucleus, while other FA proteins, such as FANCA and FANCG, may have cytoplasmic functions too.

The isoelectric point (pI) of proteins gives information about the function of proteins. Almost all FA proteins have an acidic pI, except FANCB (7.65) and FANCF (9.20) with the average isoelectric point constituting 6.5. Interestingly, the pI of interacting proteins is usually different, but together result in an optimum pH for the desired location [50], [51]. For example, the isoelectric points of the subcomplex: FANCC-FANCE-FANCF, respectively 6.09, 4.89, 9.20, result in an average pI of 6.73.

Proteins that participate in the same pathway usually display similar tissue-specific expression patterns. With GeneNote [52], it is possible to survey mRNA expression levels for a selected number of tissues. In general, the expression pattern of GeneNote profiles for FA genes ranged between normalized intensity values of ∼10–100 and was similar between the different assayed tissues, although the profile for FANCG was at the higher end of intensity. The same holds true for FANCF and FANCP/SLX4, which, also for other features, belonged to the extremes (compared to the other FA proteins, FANCF has the highest pI, and FANCP/SLX4 the highest NLS, see also Table 1). FANCF is also an exception in that it is the only one-exon FA gene.

The overall picture emerging from these characterization studies is that the vast majority of FA proteins are not highly conserved, already previous described for FANC-A, -B, -C, -D1, -D2, -E, -F, and -G [53]; NLSs - if present - are of moderate strength, proteins are acidic and the corresponding mRNAs are ubiquitously, but not highly, expressed.

### Retrieval of Information and Scoring of the Complete Proteome

The same features as queried for the FA proteins were also interrogated for the complete human proteome. Corresponding orthologous mouse-human identities were retrieved. The NLS and pI scores were calculated using the peptide sequences. In addition, other features were retrieved including whether the genes could be linked to specific annotations, such as to Gene Ontology, but also to MIM gene or MIM morbid. Furthermore, all proteins were queried against the 1) Molecular Interactions workflow of EnVision 2) the literature mining tool Nermal and 3) protein function prediction tool FuncNet. It should be noted that in principle for each protein/peptide, features, such as pI and NLS scores, can be determined, while for other queries especially when using the tools related to protein function only a subset of all proteins queried may return results.

The ranking scheme consists of two parts, one using the protein and another using retrieved literature data as starting point. The first part can be further subdivided in calculable features (NLS and pI), and other features, such as connection to Gene Ontology terms (Table 2).

**Table 2:** Scoring scheme.

Filters	Settings	Score	Remarks
***Protein***			
% ID aa and Mouse % ID	>40<85%	1.5	
Difference % ID	≤15%	2	If %ID is in the interval (40< %ID <85) and the difference between human and mouse %ID is ≤15∶0.5 point extra (2 points in total)
Peptide length	≤100 aa	–	Only peptides longer than 100 aa are scored
pI	4<pI <9	1	
NLS	≤2	1	The score is linearly decreased from 1 to 0 between an NLS increase from 2 to 5
	2< NLS ≤5	(1,0]	
GO Nucleus	Nucleus	1	Nucleus means GO accession equal to or descendant of nucleus
	Unknown	0	
	Different	−1	
Molecular Interaction (EnVision 2)	Present	1	
	Not present	0	
***Literature***			
MIM annotation	Present	1	
	Not present	0	
Nermal and FuncNet*	Present	1.5×Fisher normalized	
	Not present	−1000	

* The FuncNet and Nermal scores were integrated and a new Fisher score was calculated that includes all FuncNet P-values as well as a P-value for Nermal. The “−1000” score is merely to clearly separate entries with Nermal/FuncNet scoring from those without. The maximum score possible with this scheme is 8.5.

First, the FA protein properties described above were used to aid the scoring of the whole proteome for FA-like proteins. The settings for ranking were based on properties from the first thirteen identified FA proteins, while the most recently identified FA proteins, FANCO/RAD51C [26] and XRCC2 [29] were used for validation, and FANCP/SLX4 [27], [28] was used for fine-tuning. The percentage of identity between human and mouse protein was set between 40 and 85%. Points were given when a protein has an identity within this range. Extra weight was given if the human and mouse orthologs were more similar (i.e. when the difference in percentage identity of amino acids between human-mouse and vice versa was less than or equal to 15%). For the NLS scores, one point was given to a protein if an NLS score ≤2 was detected, which is in line with the thirteen FA proteins. However, the later identified, FANCP/SLX4, which harbours a high NLS score, would not obtain a score. To cover for this extreme, we developed a scoring in which the presence of NLS scores in the higher ranges from 2 to 5 resulted in a linear decrease in assigned points, ranging from 1 to 0. In total 94% of the peptides analysed displayed scores between −0.47 and 2.00, and therefore received points. The pI range chosen, 4<pI <9, results in 75% of the analysed proteins receiving a point. Since the relevance of a nuclear localization for the DNA repair function, the presence of the GO accession “nucleus” or a descendant of nucleus was used to give one point. In case no GO accession was available zero points were given, while one point was subtracted when the accession was not nucleus. Although the first two features evidently lack large discriminative power, these can, nevertheless, contribute to the overall scoring scheme and were therefore retained as parameter.

Second, we expanded the scoring of the proteome with publicly available interaction data. If a protein in the proteome has an interaction with at least one of the thirteen FA proteins according to the EnVision 2 Molecular Interaction results, one point was given. Further, a point was given when an OMIM entry was present; this was primarily to give known genes, including those not yet related to DNA repair pathways, a higher weight. Often, the genes were already known for a long time (e.g. RAD51C) but not yet related to FA. In addition, known genes in ranked lists can be easier ruled out/or selected for often laborious further analysis as more information is present on these genes. We also obtained scores for the tools Nermal (biosemantics; literature mining) and FuncNet (which includes the literature mining tool iHop). Nermal turned out to have a good coverage of the proteome and for a large number of proteins Nermal was the only or one of the few tools providing a prediction. However, for other proteins FuncNet was more informative. For ranking purposes, we therefore included both the Nermal and the FuncNet scores.

### Validation of the Ranking Approach

The ranking of all proteins based on FA-protein properties, resulted in a top 150 list (Table 3). The FA proteins score in general high, as can be expected since the scoring is based on their properties (Table 4). Furthermore, only 1673 unique associated gene names (including the FA proteins and proteins without a Nermal and/or FuncNet score) score 5 points or higher (Table 5) which is 7.5% of all unique associated gene names. The FANCF protein scored relatively low, compared to the other FA proteins. This could be attributed to the fact that in the automated scoring the mouse ortholog FANCF was missing, resulting in zero points for the human-mouse comparison feature. After manual correction of the percentage amino acid identity, FANCF ends up at the same level as FANCJ/BRIP1 and FANCD1/BRCA2 with 6.34 points. We hypothesized that in this particular ranking, a cut-off score ≥5 will select for FA like candidates (Table 4 and 5).

**Table 3:** Top 150 genes from the combined ranking.

Position	Gene Name	Score	Position	Gene Name	Score	Position	Gene Name	Score
1	BRCA1	8.500	51	RAD51L3	6.461	101	IFIH1	6.303
2	FANCG	8.156	52	MAD1L1	6.452	102	XPA	6.301
3	FANCA	8.131	53	BIN2	6.430	103	IRF3	6.294
4	FANCC	8.005	54	KIT	6.428	104	CDKN1B	6.293
5	RPA1	7.867	55	SMARCAL1	6.426	105	ZNF217	6.290
6	BLM	7.866	56	DBF4	6.417	106	CCNO	6.290
7	FANCD2	7.854	57	STAG3	6.416	107	PRDM2	6.284
8	BARD1	7.733	58	PML	6.408	108	RFC4	6.282
9	RECQL5	7.727	59	BRIP1	6.405	109	SPO11	6.279
10	XRCC3	7.694	60	NFE2L3	6.403	110	BCL3	6.275
11	BCCIP	7.690	61	CDKN1C	6.398	111	NUP62	6.271
12	FANCL	7.635	62	PMS1	6.397	112	ITGB3BP	6.270
13	CHEK2	7.631	63	CDC25C	6.389	113	TULP3	6.269
14	FANCE	7.618	64	CDKN1A	6.384	114	ITGAE	6.261
15	NBN	7.578	65	RAD51	6.377	115	PSMD9	6.260
16	TP53	7.554	66	OGG1	6.376	116	E2F2	6.259
17	RAD52	7.508	67	CCNA1	6.375	117	NAP1L2	6.259
18	FANCB	7.500	68	RAD51C	6.372	118	MBD4	6.259
19	MDC1	7.392	69	SUMO1	6.367	119	MLF1	6.259
20	PMS2	7.358	70	TAF1B	6.361	120	RAD54B	6.257
21	FANCM	7.311	71	RTEL1	6.361	121	IFI35	6.257
22	MUS81	7.291	72	MZF1	6.357	122	REV1	6.256
23	ATRIP	7.223	73	KNTC1	6.356	123	SRA1	6.254
24	FANCI	7.214	74	CDC7	6.354	124	ELP4	6.249
25	BRF2	7.152	75	ZWINT	6.353	125	ORC2L	6.249
26	IKBKE	7.129	76	TP53BP1	6.351	126	CDC25B	6.247
27	DNA2	7.074	77	NCL	6.349	127	MDM2	6.246
28	PALB2	6.854	78	PNKP	6.344	128	NDC80	6.243
29	BUB1	6.801	79	MAP1S	6.343	129	FOXN4	6.243
30	BACH1	6.689	80	HMOX1	6.343	130	KIF11	6.242
31	DCLRE1B	6.653	81	BRCA2	6.341	131	TAF1A	6.242
32	DDX11	6.648	82	ERCC1	6.340	132	CASP8	6.240
33	DDX12	6.648	83	SFRS1	6.338	133	ERCC5	6.240
34	CCNE1	6.568	84	ATAD2	6.336	134	WDHD1	6.240
35	RAD17	6.565	85	CHRNA4	6.335	135	NCAPD3	6.238
36	NCOA3	6.558	86	CEP250	6.330	136	RNASEL	6.238
37	RAD9A	6.550	87	APEX2	6.329	137	NCOA4	6.238
38	LIG1	6.544	88	LRPPRC	6.327	138	HIF3A	6.237
39	ERCC6	6.538	89	CHFR	6.327	139	RFX5	6.237
40	RPA3	6.526	90	USP8	6.326	140	CCNF	6.233
41	GMNN	6.502	91	RBBP8	6.325	141	KLF1	6.232
42	BUB1B	6.500	92	POLR2H	6.325	142	TFDP3	6.232
43	TTF2	6.498	93	RASSF7	6.324	143	CHEK1	6.231
44	XRCC5	6.492	94	PPARGC1B	6.322	144	DAPK3	6.228
45	XRCC4	6.488	95	TRH	6.319	145	NUP153	6.228
46	XRCC2	6.486	96	FOXM1	6.317	146	UIMC1	6.228
47	NOP14	6.469	97	PRC1	6.313	147	SOD3	6.227
48	MAPT	6.468	98	NCAPH	6.307	148	NEK3	6.226
49	ZBTB32	6.463	99	SKAP1	6.307	149	FOXO4	6.224
50	UBE2T	6.463	100	TGFA	6.305	150	UBASH3A	6.224

**Table 4:** Scores for the sixteen known FA proteins.

Established FA protein	Score
FANCG	8.16
FANCA	8.13
FANCC	8.00
FANCD2	7.85
FANCL	7.64
FANCE	7.62
FANCB	7.50
FANCM	7.31
FANCI	7.21
FANCN/PALB2	6.85
XRCC2	6.49
FANCJ/BRIP1	6.41
FANCO/RAD51C	6.37
FANCD1/BRCA2	6.34
FANCP/SLX4	5.01
FANCF	4.34*

* Manual inspection showed that the FANCF ortholog was not present in EnSEMBL (see Table 1), therefore FANCF should have scored 2 points higher.

**Table 5:** Distribution of scores in the ranked list.

Cut-off Score	# Genes
≥3.00	10610
≥4.00	7213
≥4.50	1749
≥5.00	1673
≥5.50	919
≥6.00	868
≥6.25	123
≥6.50	41
≥7.00	27

Since only the first thirteen FA proteins were used for pipeline generation, we could also determine how the three newest members of the FA protein family, FANCO/RAD51C, FANCP/SLX4, and XRCC2 were performing (Table 4). The overall score for FANCO/RAD51C and XRCC2 turned out to be in the range of known FA proteins, while FANCP/SLX4 scored lower than known FA proteins. FANCP/SLX4 has a relatively high NLS score compared to the other FA proteins, even though we adjusted the NLS score for extremes. In addition, at the time of data retrieval no OMIM entry was present for SLX4.

In the case of FA, there are strong candidates for FA genes uncovered by biochemical studies. These include the so-called FAAP proteins [30]–[33], which associate to the core complex, a nuclease FAN1 [34]–[37], and a deubiquitinating enzyme complex consisting of USP1 and UAF1 [38], [39]. These proteins, upon disruption, display the FA hallmarks including MMC sensitivity. Therefore, we were interested in how these proteins were ending up in our ranking (Table 6). Three of these, FAAP100, USP1, and FAAP24, would also have been suggested with our automated ranking scheme, with a respectively score of 6.15 (rank: 299), 5.44 (rank: 890), and 5.16 (rank: 1011). The protein UAF1 is a highly conserved in mouse (98% aa identity), and therefore had a score of 4.05 (no points for the conservation interval 40–85%). UAF1 functions as an activator of USP1 (by itself USP1 has almost no deubiquitinating activity), and the protein complex together functions as a deubiquitinating enzyme. Interestingly, it has been reported that UAF1 is an abundant protein in human cells, and that UAF1 may have additional functions [39], besides regulating the FA pathway. For the three other FAAPs (FAAP16∶4.05, FAAP20∶2.00, and FAAP10∶1.11) and FAN1 (4.53), manual correction of the orthology feature was required, as was the case FANCF. In addition, FAAP10 was not properly scored, due to the fact that the threshold for scoring of peptides was set at larger than 100 amino acids, and FAAP10 is 81 amino acids in length. Overall, the chosen settings leads to logical candidates in the top. However, awareness is important, since strong candidates may not show up by, for example, wrong mapping of orthologs.

**Table 6:** Scoring output for FA associated proteins.

		Percentage identity (aa)							
Associated protein	Score	Human-Mouse	Mouse-Human	NLS score	pI	Length* (aa)	MIM	Interaction	GO nucleus
FAAP100/C17orf70	6.15	73	61	−0.47	4.89	730	611301	–	1
USP1	5.44	88	88	1.12	5.13	785	603478	1	1
FAAP24/C19orf40	5.16	81	79	−0.22	9.70	215	610884	1	−1
FAN1/MTMR15	4.53	47	65	2.30	7.36	1017	–	–	1
FAAP16/MHF1/APITD1	4.05	N/A	N/A	0.02	7.87	164	609130	–	1
UAF1/WDR48	4.05	98	98	−0.47	7.04	677	612167	–	1
FAAP20/C1orf86	2.00	30	46	−0.47	8.71	283	–	–	−1
FAAP10/MHF2/STRA13	1.11	N/A	N/A	−0.47	5.69	81	–	–	1

* NLS and pI based on longest peptide.

The relatively large difference in percentage identity between human-mouse and mouse-human of FAN1/MTMR15 indicates a difference in sequence length. Indeed, upon manually checking the length for the human MTMR15 protein turned out to be 1017 aa (ENSP00000354497) and for the mouse ortholog 743 aa (ENSMUSP00000103138). When interrogating the FAN1 ortholog at NCBI HomoloGene, both proteins were of similar length and an identity score of 71% was predicted for human-mouse and mouse-human (Human: NP_055782.3, Mouse: NP_808561.2). The missing or wrong orthologs in EnsEMBL also contribute to the lower scoring of FAAP16 (human-mouse 78% amino acid identity; mouse-human 83% amino acid identity) and FAAP20 (human-mouse 53% amino acid identity; mouse-human 53% amino acid identity) and FAAP10 (human-mouse 64% amino acid identity; mouse-human 64% amino acid identity) when interrogating the orthologs at NCBI HomoloGene.

To further validate the method, we analyzed several proteins that are involved in DNA repair processes that the FA/BRCA pathway is coordinating, which include nucleolytic incision, translesion DNA synthesis (TLS), and homologous recombination [54] (Further see Table 7). Of the 26 analyzed proteins, 20 score above 5 points, of which 12 are also in the top 150: BRCA1, XRCC3, NBN/NBS1, RAD52, MUS81, XRCC2, RAD51L3/RAD51D, RAD51, ERCC1, RBBP8/CtIP, RAD54B, and REV1 (Table 3 and 7). REV1, is involved in translesion DNA synthesis and scores higher (6.26) than REV3 (4.22) and REV7 (3.14) that together form a complex called Pol ζ, mainly because the last two are highly conserved (more than 85%). REV1 functions as a scaffold protein to recruit and coordinate TLS polymerase, such as Pol ζ [55], [56]. Pol ζ has besides translesion DNA synthesis another essential role in cell proliferation [57]. The difference in score suggest that a high score (≥5) can distinguish between proteins that have a direct interaction with the FA/BRCA pathway (USP1 and REV1) and proteins that are coordinated via this link (UAF1 and Pol ζ).

**Table 7:** Overview proteins involved in processes coordinated by the FA/BRCA pathway.

Protein	Score	Process*
BRCA1	8.50	homologous recombination
XRCC3	7.69	homologous recombination
NBN/NBS1	7.58	homologous recombination
RAD52	7.51	homologous recombination
MUS81	7.29	nucleolytic incision
XRCC2	6.49	homologous recombination
RAD51L3/RAD51D	6.46	homologous recombination
RAD51	6.38	homologous recombination
ERCC1	6.34	nucleolytic incision
RBBP8/CtIP	6.33	homologous recombination
RAD54B	6.26	homologous recombination
REV1	6.26	translesion DNA synthesis
EME1	6.22	nucleolytic incision
EME2	6.06	nucleolytic incision
GEN1	6.05	nucleolytic incision
RAD51L1/RAD51B	5.86	homologous recombination
MRE11A	5.57	homologous recombination
DMC1	5.52	homologous recombination
ERCC4/XPF	5.32	nucleolytic incision
RAD50	5.17	homologous recombination
RAD54L	4.75	homologous recombination
REV3/REV3L	4.22	translesion DNA synthesis
GIYD2	4.00	nucleolytic incision
SHFM1	3.76	homologous recombination
REV7/MAD2L2	3.14	translesion DNA synthesis
GIYD1	3.00	nucleolytic incision

* Information based on http://sciencepark.mdanderson.org/labs/wood/DNA_Repair_Genes.html and [54].

**Figure 2:**
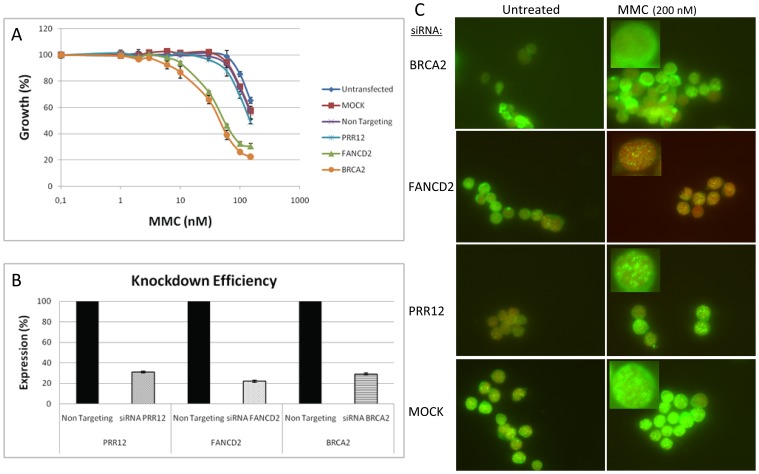
Validation of FA candidate PRR12. MMC growth inhibition assays and RAD51 foci in HeLa cells after siRNA knockdown with specific siRNAs for BRCA2, FANCD2, and PRR12 or with negative control siRNAs. A) MMC growth inhibition assays after siRNA knockdown of BRCA2 and FANCD2 show sensitivity to MMC. siRNA knockdown of PRR12 does not result in MMC sensitivity. Data represent average values from one representative experiment performed in triplicate. B) Relative gene expression was calculated via the 2^−ΔΔC^T method, normalized against the non targeting oligo and *TBP.* The normalized expression (average of two different primer sets per gene) of the non targeting oligo was set at 100%, and knockdown efficiency for BRCA2, FANCD2, and PRR12 siRNA has been indicated. C) siRNA knockdown of BRCA2 results in diminished RAD51 foci, whereas siRNA knockdown of FANCD2 and PRR12 show RAD51 foci after knockdown. Green: αRAD51; Red: αTOPRO3.

In addition, we investigated a prime candidate for a novel FA gene based on whole-genome next generation sequencing data. The particular gene, *PRR12*, harboured two possible pathogenic mutations, which followed proper segregation as expected for a recessive disorder (unpublished data). However, based on our ranking system, this gene turned out to be an unlikely candidate, having only 1 point. Further functional testing, confirmed that PRR12 did not display the hallmark FA features. Upon *PRR12* disruption by siRNA, the cells were still resistant to MMC. Furthermore, downregulation of PRR12 did not result in a reduction of RAD51 focus formation (Figure 2; Material and Methods S1), which is characteristic for the cells of the patient in which the *PRR12* variants were found (unpublished data). This confirmed that a low score in our ranking corresponded to a non-FA protein (in this case).

**Figure 3:**
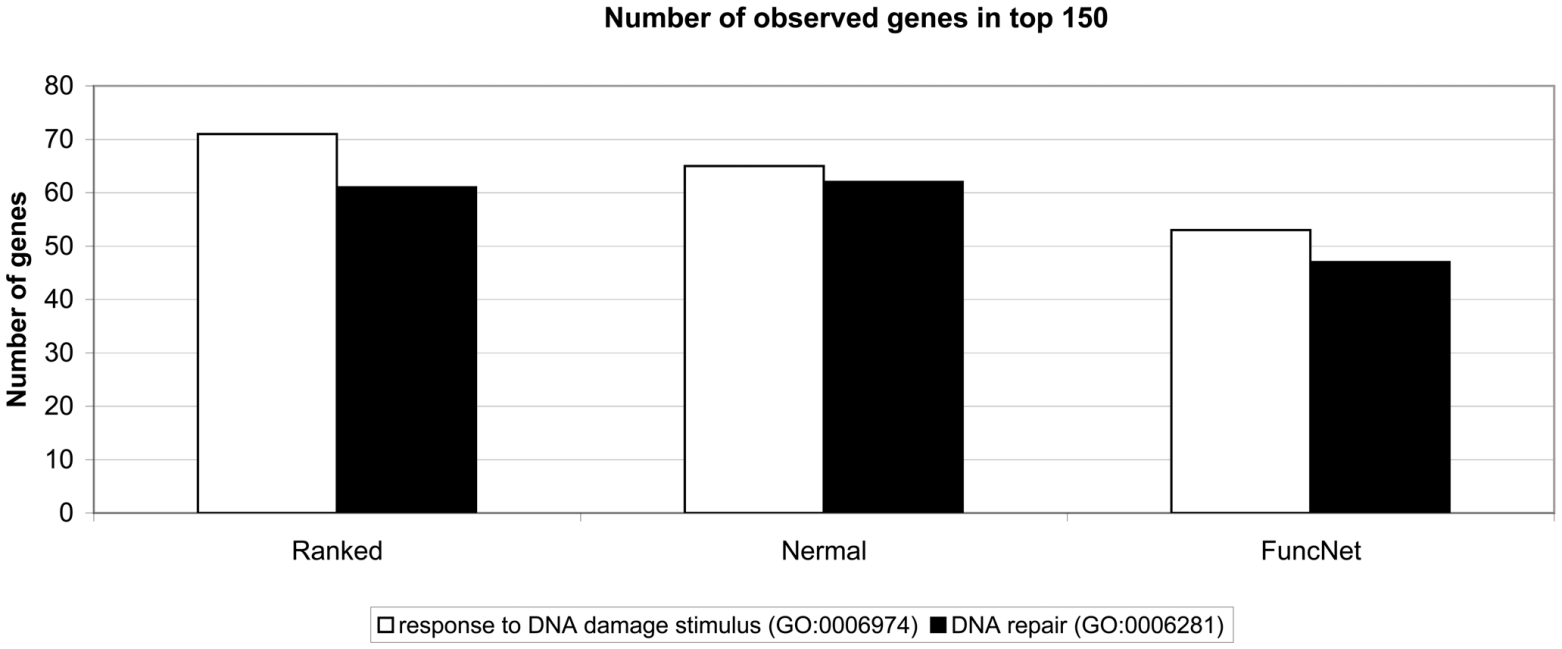
GO enrichment top 150 of Ranked, Nermal, and FuncNet. Number of genes observed with the GO terms “response to DNA damage stimulus” (GO:0006974) and “DNA repair” (GO:0006281) in the top 150 of our combined ranking approach “Ranked”, the literature mining tool “Nermal”, and the protein function prediction tool “FuncNet”. Number of genes observed, number of genes expected (P-value) for GO term “response to DNA damage stimulus” (GO:0006974): Ranked 71, 5 (5.50E−64); Nermal 65, 5 (7.10E−56); FuncNet 53, 5 (4.27E−42). GO term “DNA repair” (GO:0006281): Ranked 61, 4 (1.28E−60); Nermal 62, 3 (1.24E−62); FuncNet 47, 3 (1.54E−42).

**Figure 4:**
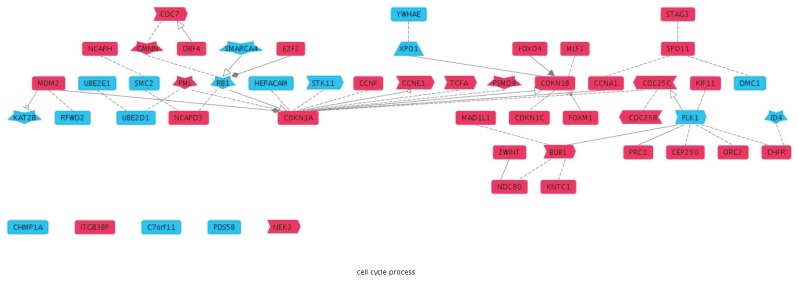
Overview genes involved in “Cell cycle process”, Ranked vs Nermal. Analysis of the overlap between our combined approach and Nermal for the GO biological process term “Cell cycle process” (GO:0022402). Top 150 of either our combined ranking or Nermal alone were analyzed for GO term “DNA repair” (GO:0006281) and these genes were discarded. The remaining lists (Ranking combination scheme: 89 genes and Nermal: 88 genes) were further compared. In total, 7 genes were found in common (*BACH1*, *NFE2L3*, *DDX11*, *CHEK2*, *MAPT*, *BUB1B*, *UBASH3A*). A combined list of the remaining genes (Ranking: 81 genes and Nermal: 82 genes; total 163 genes) was analyzed with the Genomatix Pathway System (GePS). The biological process term “Cell cycle process” was the most enriched (P-value: 4.83E−25). The relation between the different cell cycle proteins is depicted (red: candidates combined ranking scheme, blue: candidates Nermal).

Furthermore, we evaluated the performance of the ranking scheme for identification of proteins enriched for functional classifications relevant to FA. A clear enrichment was found when screening the top 150 of the list for GO annotations “response to DNA damage stimulus” (GO:0006974; observed: 71; expected: 5; P-value: 5.50E−64) and “DNA repair” (GO:0006281; observed: 61; expected: 4; P-value: 1.28E−60; Figure 3). To demonstrate the effect of our combined scoring using multiple criteria, we also compared our top 150 list (“Ranked”) to the top 150 lists of the literature-mining tool “Nermal” and the protein function prediction tool “FuncNet”. When only FuncNet was used, the number of genes with the GO annotation for “response to DNA damage stimulus” (observed: 53; expected: 5; P-value: 4.27E−42) and “DNA repair” (observed: 47; expected: 3; P-value: 1.54E−42) were clearly lower compared to our ranking. However, the top 150 of Nermal “response to DNA damage stimulus” (observed: 65; expected: 5; P-value: 7.10E−56) and “DNA repair” (observed: 62; expected: 3; P-value: 1.24E−62) results in similar number of genes for the “DNA repair” annotation (Ranked: 61 genes and Nermal: 62 genes). To study the difference between our ranking (including Nermal and FuncNet) and Nermal alone, we also analyzed the remaining genes in the top 150 that do not have the “DNA repair” annotation for functional categories (Table 8 and 9). We chose for the “DNA repair” term so that both lists are comparable in size (for Ranked: 150 genes –61 genes with GO term “DNA repair” and 89 genes that do not have the GO term “DNA repair” and for Nermal: 150 genes –62 genes with GO term “DNA repair” and 88 genes that do not have the GO term “DNA repair”). In the case of our approach, the remaining genes were significantly enriched for cell cycle-regulated genes (10 most significant categories; all cell cycle; Table 8). The emphasis on cell cycle parallels the important role for FA proteins in the cell cycle. From the top 10 GO enrichments obtained for Nermal, only 4 GO terms were cell-cycle related (Table 9). The relation between the different cell-cycle related proteins uncovered by our approach (including Nermal and FuncNet) or with Nermal alone is shown in Figure 4.

**Table 8:** Top 10 biological processes for the combined ranking.

			# Genes		
GO-Term	GO-Term id	P-value	Observed	Expected	Total
cell cycle process	GO:0022402	1.24E−23	38	5.33	888
cell cycle phase	GO:0022403	4.00E−23	35	4.41	735
mitotic cell cycle	GO:0000278	2.62E−22	33	3.98	663
cell cycle	GO:0007049	2.33E−21	40	7.04	1173
regulation of cell cycle arrest	GO:0071156	8.52E−16	18	1.34	223
regulation of cell cycle	GO:0051726	3.32E−15	25	3.51	585
cell cycle arrest	GO:0007050	4.27E−15	20	1.97	329
regulation of cell cycle process	GO:0010564	5.08E−15	20	1.99	332
negative regulation of cell cycle	GO:0045786	6.90E−15	21	2.31	385
cell cycle checkpoint	GO:0000075	7.67E−15	17	1.28	214

Biological process enrichment for the 89 remaining genes of the ranked top 150 that do not have the GO annotation “DNA repair” (GO:0006281). The 89 genes were analysed for enrichment via Genomatix GeneRanker. # Genes Observed = the number of genes from the input set which have this annotation; # Genes Expected = the number of genes one would expect to have this annotation based on the input set; # Genes Total = the number of genes from the complete genome which have this annotation.

**Table 9:** Top 10 biological processes for Nermal.

			# Genes		
GO-Term	GO-Term id	P-value	Observed	Expected	Total
negative regulation of cellular process	GO:0048523	1.10E−11	38	12.18	2104
negative regulation of biological process	GO:0048519	1.62E−10	38	13.30	2298
cell cycle	GO:0007049	2.66E−08	24	6.79	1173
cell proliferation	GO:0008283	4.81E−08	24	7.00	1210
cell cycle process	GO:0022402	1.10E−07	20	5.14	888
protein modification by small protein conjugation or removal	GO:0070647	1.34E−07	14	2.46	425
regulation of cell proliferation	GO:0042127	1.47E−07	20	5.23	904
regulation of cell cycle	GO:0051726	1.96E−07	16	3.39	585
protein modification by small protein conjugation	GO:0032446	2.20E−07	13	2.17	375
negative regulation of cell cycle	GO:0045786	2.97E−07	13	2.23	385

Biological process enrichment for the 88 remaining genes of the Nermal top 150 that do not have the GO annotation “DNA repair” (GO:0006281). The 88 genes were analysed for enrichment via Genomatix GeneRanker. # Genes Observed = the number of genes from the input set which have this annotation; # Genes Expected = the number of genes one would expect to have this annotation based on the input set; # Genes Total = the number of genes from the complete genome which have this annotation.

Proteins that participate in the same pathway usually display similar expression. As mentioned above, FA genes are expressed in a variety of tissues, but not at very high levels (*cf*. GeneNote profiles). The expression of a subset of mRNAs for FA genes is downregulated upon serum withdrawal and, although less strongly, upon reaching confluence, as for example tested in the human T98G cell model [58]. Genes such as *DNA2*, *BUB1*, *DCLRE1B*, *DDX11* and *GMNN* from the top 150 list are similarly regulated for these cell cycle features and may display an as yet not recognized interaction with the FA network.

**Figure 5:**
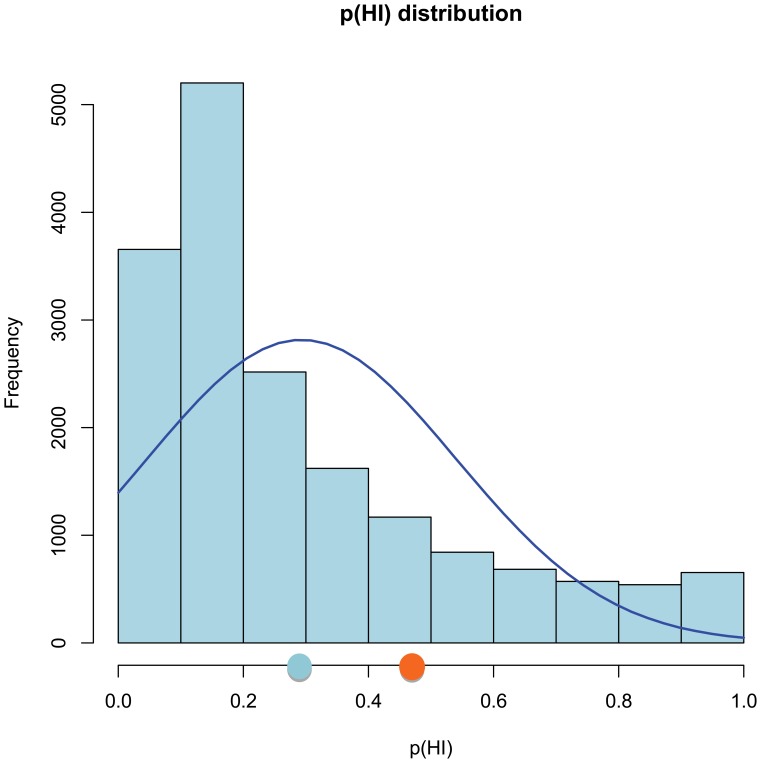
Distribution of haploinsufficiency probabilities (p(HI)). Distribution of haploinsufficiency probabilities (p(HI)) for the human genome (from Huang *et al*. [61]). The mean p(HI) values for all human genes and 143 genes of our top 150 set are given as respectively, light blue and orange circles on the x-axis. p(HI)  = 1 means highly haploinsufficient, p(HI)  = 0 haplosufficient.

**Figure 6:**
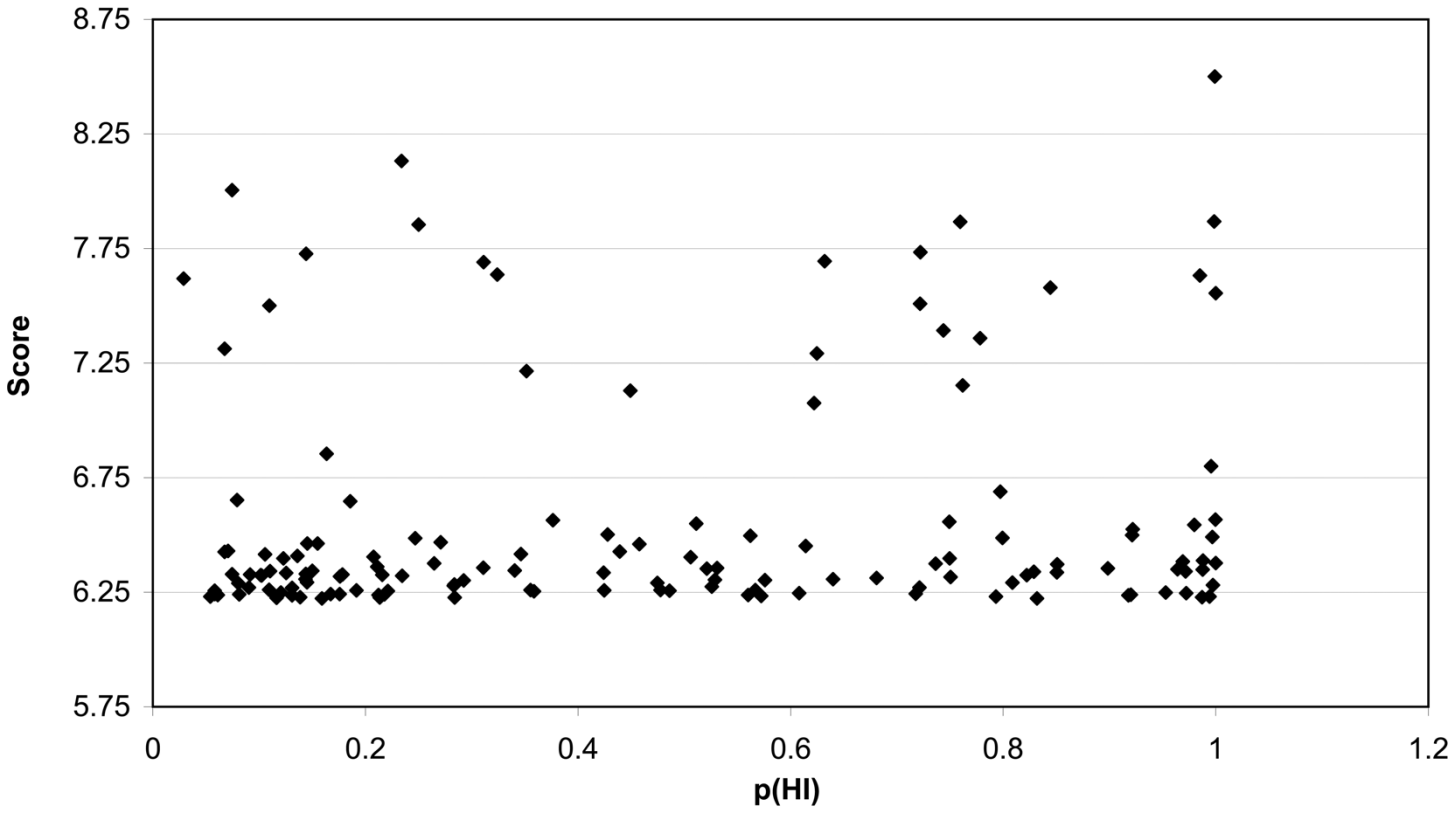
Distribution of scores of the top 150 vs p(HI). The scores of the top 150 of our ranked approach were plotted against the p(HI) values of Huang et al. [61]. Shown are 143 genes, since 7 genes were not available in Huang’s data set.

So far, only biallelic/recessive mutations in the genes of the upstream branch (FANC-A, -B, -C, -E, -F, -G, -L, and -M) cause a clinical phenotype. The downstream branch, harbouring *FANCD1/BRCA2*, *FANCN/PALB2*, and *FANCJ/BRIP1*, both biallelic and/or monoallelic mutations result in a disease phenotype, notably breast cancer predisposition [59]. The top candidate in our ranking *BRCA1*, is another breast cancer predisposition gene. Interestingly, recently biallelic deleterious *BRCA1* mutations were discovered in a 28 year-old woman diagnosed with ovarian cancer, who was extremely sensitive to DNA interstrand cross-linking chemotherapy [60]. Haploinsufficiency defines the state of a given gene where a single copy is insufficient to maintain normal function, which is one of the major causes of dominant diseases. We used recent data from Huang *et al*. (2010 [61]) to estimate the haploinsufficiency probability p(HI) in our top 150 genes. A p(HI) of 1 indicates high probability of haploinsufficiency, while 0 indicates haplosufficiency. We could determine p(HI) values for 143 genes resulting in a mean probability of 0.45 which is significantly different from the mean of 0.29 for the whole human set for which p(HI) could be predicted (Figure 5). To determine how the p(HI) is distributed over the 143 genes in our top 150 we plotted these against each other (Figure 6) and both genes with low and high p(HI) were detected, as expected. When comparing the group of genes with both a high ranking score (>6.75), and with almost opposite p(HI) values (<0.2 versus >0.9), four FA genes ended up in the first group, while established breast cancer predisposition genes ended up in the latter group. However, PALB2 has a low p(HI) value (Table 10). Interestingly, the five genes with a ranked score above 6.75 and a p(HI) value above 0.9 (*TP53*, *BRCA1*, *RPA1*, *BUB1*, and *CHEK2*) have Medical Subject Heading (MeSH) terms for small cell carcinoma (C04.557.470.200.380) and glioblastoma (C04.557.580.625.600.380.080.335, C04.557.470.670.380.080.335, C04.557.465.625.600.380.080.335). These data suggest that by overlaying the p(HI) score the top 150 can be divided in two groups, one containing the upstream branch and related genes, and the other group with the downstream branch related genes.

**Table 10:** High scoring genes versus low (<0.2) and high (>0.9) p(HI).

Score Ranking >6.75	
p(HI) <0.2	p(HI) >0.9
PALB2	TP53
RECQL5	BRCA1
FANCB	RPA1
FANCC	BUB1
FANCM	CHEK2
FANCE	

### Application of Prioritization Approach

Ranking tools can be valuable to sort/sift through candidates obtained through whole exome sequencing experiments. In principle, the identification of a causal gene in the case of a recessive disorder, such as Fanconi anemia, is straightforward; the causal gene should harbour two pathogenic mutations (1 mutation in both copies). In practice, the situation may be more cumbersome. Genes harbouring two possible pathogenic mutations may turn out to be not the cause of the disease (see also above), while genes for which only one mutation has been reported may in fact require further study due to the fact that mutations could be missed owing to imperfections in the sequencing procedure, such as lack of coverage or complex mutation types not detectable by the technique. Lists of genes harbouring only one mutation will be inevitably significantly longer than lists of genes harbouring two mutations. Sifting through these lists may be significantly aided by specially tailored ranking tools which should be combined with manual curation.

### Conclusion

We show how we used publicly available bioinformatics tools and databases to generate ranked lists tailored for FA-like genes. While the tools by themselves can be discriminative, an integrative approach exploiting multiple intrinsic features of FA proteins combined with functional and text mining resources may result in lists that are highly enriched for proteins of the network of interest. This kind of prioritization can be useful for Next Generation Sequencing projects to prioritize possible disease genes from an extensive list of candidates. Furthermore, since some of the FA proteins have been identified as genes involved in breast cancer we believe that our prioritization strategy can also be applied in studies to screen for breast cancer predisposition genes.

## Supporting Information

Material and Methods S1 (DOC)
