# Correction: A Protein Prioritization Approach Tailored for the FA/BRCA Pathway

**DOI:** 10.1371/annotation/bd2ee3f6-0f2b-4814-9825-7b90a40fb91f

**Published:** 2013-10-15

**Authors:** Anneke Haitjema, Bernd W. Brandt, Najim Ameziane, Patrick May, Jaap Heringa, Johan P. de Winter, Hans Joenje, Josephine C. Dorsman

There was an error in the Funding Statement. The Funding Statement should read: “This work was supported by ENFIN, a Network of Excellence funded by the European Commission within its FP6 Programme, under the thematic area 'Life sciences, genomics and biotechnology for health' (contract number LSHG-CT-2005-518254). The funders had no role in study design, data collection and analysis, decision to publish, or preparation of the manuscript.” 

