# Supplementary material for: A Protein Prioritization Approach Tailored for the FA/BRCA Pathway
Source: PLoS One. 2013 Apr 19;8(4):e62017. doi: 10.1371/journal.pone.0062017 (PMC3631253; doi:10.1371/journal.pone.0062017)
Supplement: Material and Methods S1 — (DOC) [file pone.0062017.s001.doc]

**Material and Methods S1**

**HeLa S3 cell culture**

HeLa S3 cells were cultured in Dulbecco’s modified Eagle medium (DMEM) containing 4,5 g/L D-Glucose supplemented with 10% fetal bovine serum (FBS).

**siRNA experiments**

Knockdown experiments were performed in HeLa cells. We used siGENOME SMARTpool siRNAs (Thermo Scientific) against *BRCA2*, *FANCD2*, *PRR12* and non-targeting. Knockdown experiments were performed with Lipofectamine® 2000 transfection reagent (Invitrogen) according to the manufacturer’s protocol. Seventytwo hr after transfection, the cells were divided over 4-well glass slides (5,000-10,000 cells/well), 96-well plates (750 cells/well) and the remainder of the cells were collected for total RNA isolation (see RNA extraction, cDNA synthesis and qRT-PCR analyses).

**MMC growth inhibition assay**

MMC was added in various concentrations (0, 1, 2, 3, 6, 10, 30, 60, 100, and 150 nM) to the 96-wells plate containing the HeLa S3 cells transfected with siRNAs. After 4 days of MMC treatment, cell viability was measured with CellTiter-Blue Cell Viability assay. Percentage growth was calculated compared to the untreated cells.

**Immunofluorescence**

Cells were grown on 4-well glass slides and treated with 200 nM MMC for 24 hrs prior to immunofluorescence. Cells were washed twice with PBS, and shortly treated with 0,25% Triton X-100 on ice before fixation with 4% paraformaldehyde for 15 min at room temperature, subsequently cells were permeabilized with 0,5% Triton X-100 for 20 min and blocked for 1 h at room temperature in blocking buffer (10% FBS in PBS). The slides were incubated for 2 hr at room temperature with a rabbit anti-RAD51 antibody (kindly provided by prof. dr. R. Kanaar, Erasmus University Rotterdam, The Netherlands) diluted in blocking buffer. After 4 washes with 0,2% Triton X-100 in PBS the slides were incubated with fluorochrome-conjugated secondary antibodies anti-rabbit ALEXA488 (1:1000, Invitrogen) and TO-PRO®-3 iodide (1:1000, Invitrogen) for 1 hr at room temperature. Finally, slides were washed 4 times with 0,2% Triton X-100 in PBS and embedded. Slides were analyzed with a fluorescent microscope (DM5000, Leica).

**RNA extraction, cDNA synthesis and qRT-PCR analyses**

Total RNA was extracted (High Pure Isolation Kit; Roche) and cDNA was prepared (iScript cDNA Synthesis Kit; Biorad). The mRNA levels were quantified by real-time quantitative polymerase chain reactions (SYBRGreen reaction kit; Lightcycler 480, Roche).

Per gene, two primer sets were used:

BRCA2 (5’-CCAAGTCATGCCACACATTC-3’, 5’-GGAGTGCTTTTTGAAGCCTTT-3’ and

5’-CAGCCCAGTTTGAAGCAAAT-3’, 5’- TCAGAATTGTCCCAAAAGAGC-3’)

FANCD2 (5’- TCCGACTTGACCCAAACTTC-3’, 5’- GTGATGGCAAAACACAATGC-3’ and

5’- AAGAATTTGTTAGTGGCCTGGA-3’, 5’- ATGTCAATCCCCAGAAGCAG-3’),

PRR12 (5’- GGAGTTACGAGAGGTCAGCG-3’, 5’- AGGCCAGTGTCGAAGAGTC-3’ and

5’- CGAGGCCGGGGTCGAAAGG-3’, 5’- CAGGCTGTTGTGGTCAGTGCC-3’).

Relative gene expression was calculated via the 2^-ΔΔC^T method, normalized against non targeting and *TBP* (5’-TGCACAGGAGCCAAGAGTGAA-3’, 5’-CACATCACAGCTCCCCACCA-3’). Knockdown efficiency was calculated comparing the siRNA knockdown to the negative control (non-targeting).
